# Techno-economic analysis of an HRES with fuel cells, solar panels, and wind turbines using an improved Al-Biruni algorithm

**DOI:** 10.1016/j.heliyon.2023.e22828

**Published:** 2023-11-26

**Authors:** Bofan He, Nurlida Ismail, Kimberley Khoo Kim Leng, Gang Chen

**Affiliations:** a School of International Business, Zhejiang Yuexiu University, Shaoxing 312000, Zhejiang, China; b School of Management & Marketing, Taylor's University, Subang Jaya 47500, Selangor, Malaysia

**Keywords:** Hybrid green energy system, Al-Biruni's algorithm, Optimization, Photovoltaic, Energy storage, Fuel cells

## Abstract

This research demonstrates the application of novel optimization methods in the realm of renewable energy and contributes to the development of environmentally friendly electricity generation and consumption. In this study, an improved version of the Al-Biruni algorithm has been proposed for Hybrid Renewable Energy Systems (HRES) optimization, which includes fuel cells, photovoltaic cells, and windmills. The algorithm considers supply, demand, and energy storage constraints and seeks the best combination of energy sources to meet load demand while reducing total system cost. Inspired by ancient Iranian philosopher Abu Biruni, the proposed method includes modifications to explore solution space efficiently and improve answer value. The proposed HRES model is applied to a case study from Dunhuang City, China, and its findings are validated by comparing it with other optimization approaches. The Modified Al-Biruni Earth Radius (MBER) algorithm is found to be the most efficient and reliable system, costing 4.23 million units of currency. Compared to other optimization approaches, MBER exhibited a total cost of 4.1 million US dollars, 0.009, 3.7, 3.7, LPSP, and 356 h per year. The overall cost is 5.26 million units of currency with a 0.5% Loss of Power Supply Probability (LPSP), which directly impacts system performance and dependability. The improved Al-Biruni algorithm can efficiently optimize the system, reduce costs, and increase load supply, contributing to the growth of renewable energy sources and the application of advanced meta-heuristic techniques in complex energy systems.

## 1 Introduction

The development of sustainable energy systems is driven by increasing global demand for electricity and minimizing CO2 emissions [1]. Integrated clean energy systems have gained popularity among these systems due to their capacity to provide reliable and efficient electricity while reducing environmental impact [2]. These systems combine many renewable energy technologies, including fuel cells, photovoltaic cells, and windmills to produce a more stable and reliable energy source [3]. Integrated green energy systems have grown in popularity in recent years due to the potential for sustainable, efficient, and environment-friendly production of electricity [4]. Individual energy sources have certain limitations, but integrated systems may exceed these limitations and continue to provide energy even when one source is unavailable. In addition, integrated systems can save costs, increase sustainability, and increase overall energy efficiency [5].

Integrated sustainable energy systems have the ability to minimize carbon dioxide emissions and reduce global warming. Non-renewable energy sources, such as fossil fuels, have been a major concern for the environment [6]. The use of sustainable energy sources, such as hybrid sustainable energy systems, can help reduce carbon dioxide emissions and promote sustainability. Integrated systems are also critical to mitigating global warming and supporting environmental sustainability. Photovoltaic panels are suitable for use in sunny areas because they convert solar radiation into electricity [7]. Ideally suited for windy locations, wind farms convert the kinetic energy of the wind into energy. Fuel cells generate energy by converting hydrogen or other fuels and are suitable for use in places without access to the power grid. Integrated sustainable energy systems can provide a more reliable and sustainable energy supply by integrating these multiple renewable energy sources [8].

These systems' complex interactions among various energy sources, storage systems, and load needs make it difficult to optimize their efficiency [9]. Recently, meta-heuristic optimization approaches have been considered as a potential way to enhance the effectiveness of hybrid green energy systems, focusing on cost reduction and load provisioning. Optimizing the efficiency of integrated renewable energy sources is a complex and difficult task [10]. The effectiveness of these types of systems is determined by various variables, such as the availability of clean energy sources, the effectiveness of energy conversion, the potential of energy storage devices, and electricity demand [11]. The relationships among these parameters may be complex, making it challenging to improve the system's effectiveness. In addition, the volatility of sustainable energy sources, including wind and solar energy has an impact on the effectiveness of combined renewable energy systems [7]. To optimize the efficiency of combined sustainable power systems, improved algorithms that can control these complicated relationships and discover optimal solutions that balance the trade-offs between cost, accuracy, and environmental effect, must be developed [12]. These mathematical models should be able to analyze massive volumes of data and make assessments in real time [13].

Meta-heuristic algorithms, machine learning, and artificial intelligence are advanced optimization approaches that can enhance the efficiency of integrated sustainable energy systems. They can also facilitate the integration of energy storage devices that can store excess energy produced by renewable sources and release it when needed [14]. Meta-heuristic optimization techniques are a type of optimization method that has become more important due to its ability to solve complex optimization problems. They are based on metaheuristic search approaches that replicate the behavior of natural systems and use a trial-and-error approach to find the best solution without the need for a formal computational description. Meta-heuristic optimization methods are classified into several categories, each of which has its own approach to solving optimization problems. These strategies are intended to solve problems by exploring the search space and discovering the best answers [15].

Metaheuristic optimization approaches have been effectively applied to a wide range of optimization issues, including those in the field of sustainable energy systems. These methodologies have been utilized for optimizing the planning and operation of sustainable energy sources, such as wind turbines, solar panels, and fuel cells [16]. These strategies can assist to enhance the efficiency, dependability, and cost-effectiveness of these systems; moreover, it can minimize the complexity of optimization issues, increase the pace of the development of sustainable energy sources, and enhance sustainability [17].

Several research examined that how metaheuristic optimization approaches may be used to improve the effectiveness of hybrid sustainable power systems. As an example,

Güven et al. [1] employed the combined metaheuristic optimization methodologies to analyze the efficiency of an automated renewable energy system. This study was based on financial and technological aspects of wind, solar, biomass gasifier, and off-grid fuel cells. The main objective of the optimization process was to manage the power supply between different parts of a microgrid to reduce the annual costs of the system. The Hybrid Firefly Genetic Algorithm (HFGA) was used to determine the optimal size of each component. The results showed that the proposed stand-alone hybrid energy system (PV/WT/BG/FC) was the most cost-effective alternative for the central university campus selected as the research site, and the proposed method had great convergence and convergence potential.

Bo et al. [2] proposed the ideal structure of a hybrid wind, solar panel, and fuel cell system based on an updated version of the Dragon Fly optimization algorithm. In order to electrify a rural area in Turkey, this study proposed a kind of Hybrid Renewable Energy System (HRES) that combines a solar system, a wind turbine, and a fuel cell. A modified version of the DragonFly optimizer was used to generate the system's size. The simulation results showed that among the alternatives, the proposed method provided the lowest net present cost value with $1,888,827.5. The main goal was to evaluate the objective function by minimizing the Net Current Cost (NCC) with verification based on the Loss of Power Supply Probability (LPSP). The results of the final simulations showed that the proposed method had lower NPC and LCOE than the alternative methods.

Lei et al. [3] minimized the cost of electricity production for a grid-connected integrated green energy system by utilizing the modified seagull optimization approach. This study examined an integrated renewable energy system composed up of fuel cells, photovoltaic cells, wind turbines, inverters, rectifiers, and Electrolyzers. The suggested modified seagull optimization approach was used to determine the ideal size of each component to obtain the lowest possible cost for electricity generation. A practical investigation using the suggested system's hybrid power and optimization was carried out in Qingdao, China. In comparison with the traditional SOA and MFFA approaches, the suggested approach produced results that were 2.02% and 2.78% better and converged 69.36% and 47.07% faster, respectively. The combined system might also function with great dependability and a loss of electric power probability that was substantially below the MFFA threshold.

A combined solar-wind-hydrogen CHP system suited to residential use optimized through effective metaheuristic methods by Maleki et al. [4]. The most important features in this paper were the financial framework and optimization approach for the efficiency of a grid-connected hybrid combined heat and power systems for households that employed PV, wind, and fuel cell techniques. The optimization goal was to reduce the implementation and maintenance costs of hybrid combined heat and power plants while keeping relevant restrictions in mind. The results demonstrated that the grid-connected hybrid combined heat and power approach would be the most cost-effective for meeting household load in the near future, with the genetic algorithm outperforming the Particle Swarm Optimization method.

Vatankhah et al. [5] suggested the Modified Gray Wolf Optimization method was used to create an optimally sized wind, solar energy, and fuel cell's off-grid hybrid energy plant. The main goal of the research was to reduce energy production costs while meeting system dependability limitations. The Gray Wolf Optimization algorithm was employed to optimize the system, a hybrid metaheuristic algorithm based on the modified-gray wolf optimization algorithm, and Particle Swarm Optimization algorithms was presented. The results showed that the Gray Wolf Optimization algorithm generated higher optimal outcomes than the well-known Particle Swarm Optimization algorithm, and that the created hybrid approach outperformed both the Gray Wolf Optimization and Particle Swarm Optimization algorithms.

The main novelty of this study lies in the introduction of an enhanced version of the Al-Biruni algorithm for the optimization of Hybrid Renewable Energy Systems (HRES) that comprise fuel cells, photovoltaic cells, and wind turbines. The proposed algorithm considers supply, demand, and energy storage constraints, aiming to identify the optimal combination of energy sources to meet load demand and minimize the total system cost. The enhancements made to the Al-Biruni algorithm have improved its efficiency in exploring the solution space and providing more valuable insights. By accurately predicting electricity production and consumption trends, the algorithm enables better utilization of available energy resources and reduces waste. This optimized HRES design offers a sustainable and cost-effective energy supply, reducing reliance on conventional fossil fuels. By applying advanced meta-heuristic techniques to complex energy systems, this study contributes to the advancement of renewable energy sources. It underscores the potential of the improved Al-Biruni algorithm in optimizing HRES, enhancing load supply, reducing costs, and minimizing carbon dioxide emissions, thereby improving air quality.

## 2 System modeling

### 2.1 System under study

The given research attempts to create an integrated system that combines several renewable energy sources to satisfy a certain load demand. A Photovoltaic (PV) system, a Fuel Cell (FC), and Wind Turbines (WTs) are all part of the system. The PV system absorbs and transforms solar energy into electricity, whereas the FC system uses hydrogen as a fuel to create power via an electrochemical process. The WTs produce power using wind energy.

The research focuses on improving the integrated system's architecture to guarantee that it can fulfill load requirements while decreasing total energy production costs. To accomplish this goal, the researchers employed an optimization technique to calculate the ideal equipment capacity of each system component [18]. The optimization algorithm considers a variety of criteria, including energy demand, renewable energy availability, and energy production costs.

In the examined system, the FC system incorporates a hydrogen saving tank and an Electrolyzer, allowing the system to store surplus energy generated by the PV system and WTs as hydrogen. When renewable energy sources are scarce, the hydrogen may be utilized as a fuel to create power in the FC system.

**Fig. 1:**
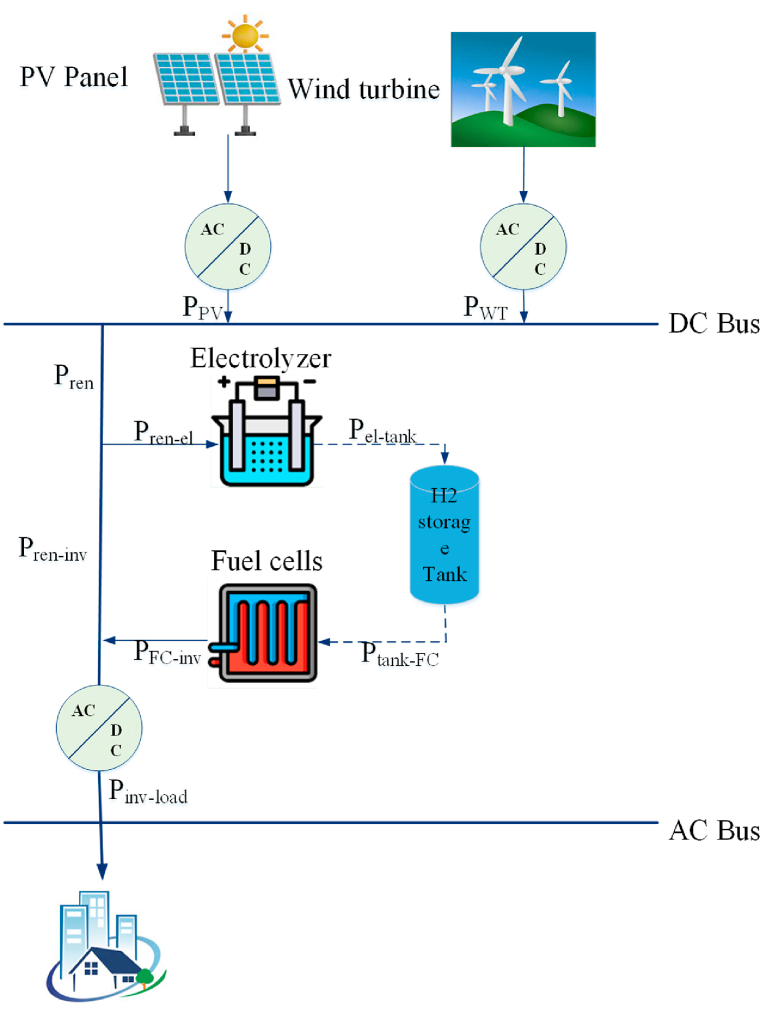
Schematic depiction of the investigated system, displaying the various components and how they are linked.

Fig. 1 depicts a schematic depiction of the investigated system, displaying the various components and how they are linked.

The PV system and WTs are shown, linked to a Power Conditioning Unit (PCU), which is in charge of transforming the electricity generated by renewable energy sources into a form that can be consumed by the load. The FC system is, likewise, linked to the Power Conditioning Unit (PCU), ensuring that the power generated by the FC system matches the load demand.

### 2.2 Photovoltaic system

Sunlight is an important renewable energy source that may be transformed into electrical power via a photovoltaic system. This system comprises photovoltaic cells in solar panels that are meant to convert sunlight directly into electrical power. The photovoltaic effect refers to the process of transforming sunlight into energy, using a photovoltaic system [19]. Photovoltaic systems are becoming more popular as a way to reduce greenhouse gas emissions and promote sustainable energy. This is due to the fact that photovoltaic systems create power without emitting any pollutants or emissions, making them a clean and renewable source of energy.

The quantity of electrical power that a photovoltaic system can create in a particular time period (t) is governed by a number of parameters, particularly the amount of sunlight available, the performance of the solar cells, and the size of the system. Equation (1) may be used to compute the output power of a photovoltaic system:

(1) $$P_{PV}\left(t\right)=A_{PV}\times \eta_{PV}\times G$$

where, $A_{PV}$ represents the area of the photovoltaic panels (in $m^{2}$), $\eta_{PV}$ represents the efficiency of the photovoltaic cells, and G describes the solar irradiance, which is a measure of the amount of sunlight that is available (in $W/m^{2}$).

According to the equation, the output power of a photovoltaic system is directly proportional to the area of the solar panels, the efficiency of the photovoltaic cells, and the amount of available sunshine. As a result, improving photovoltaic system design to optimize energy output while reducing costs is a key concern in the advancement of solar energy technology.

### 2.3 Wind turbine (WT)

Wind Turbines (WTs) are a sort of renewable energy technology that converts wind energy into electrical energy. This procedure makes use of a rotor that is linked to a generator. When the wind blows, the rotor rotates, allowing the generator to create energy [20].

The quantity of electrical energy that a WT can create is determined by a number of parameters, including wind speed, rotor size, rotor design, and the generator's efficiency. Equation (2) can be used to compute the work done by a WT:

(2) $$P_{WTG}=\left\{ \begin{array}{c} 0v<v_{cut}^{in}\\ \begin{array}{c} \frac{P_{r}\times \left(v^{3}-v_{ci}^{3}\right)}{v_{r}^{3}-v_{ci}^{3}}v_{cut}^{in}\leq v\leq v_{r}\\ P_{r}v_{r}\leq v\leq v_{cut}^{out}\\ 0v>v_{cut}^{out} \end{array} \end{array}\right.$$

where, $v_{r}$, $v_{cut}^{out}$ and $v_{cut}^{in}$ represent, in turn, the rated speed of the turbine, the speed of cut-out, and the speed of cut-in wind.

Furthermore, the resulting energy for a Wind Turbine has been mathematically expressed as follow equation (3):

(3) $$P_{WT}=P_{WTG}\times A_{WT}\times \eta_{WT}$$

where, $\eta_{WT}$ signifies the WT generator's efficiency, and $A_{WT}$ designates the swept area.

This means that even a slight increase in wind speed can result in a considerable increase in the quantity of electrical energy generated by the WT. The equation, however, demonstrates that the quantity of work done is controlled by the size and efficiency of the rotor and generator. As a result, improving the design of the WT to optimize energy output while reducing costs is a crucial factor in wind energy technology development.

### 2.4 Power produced by wind turbines and solar system

The total energy generated by renewable units, such as solar and wind turbines is determined by a variety of factors, including the size and capacity of the renewable units, the efficiency of the technology utilized, and the availability of the renewable resource. In general, the total energy generated by renewable units, such as solar and wind turbines is determined by a number of parameters, and improving the design and operation of these systems is critical for maximizing energy output. Renewable energy sources have the potential to become a major source of clean and sustainable energy in the future with sustained study and development.

The production power of renewable units is characterized by their availability as a function of their operation likelihood [Eq. (4]):

(4) $$P_{RE}=\left(N_{WT}-n_{WT}^{F}\right)\times P_{WT}+\left(N_{PV}-n_{PV}^{F}\right)\times P_{PV}$$

where, $N_{PV}$ and $N_{WT}$ represent the number of the PV arrays and wind turbines, and $n_{PV}^{F}$ and $n_{WT}^{F}$ describe, in turn, the number of damaged PV arrays and WTs.

The difference between damaged and healthy units is written as unit availability. Eq. (4) declares the idea of facility obtainability and what is referred to as the potential of their action, as well as the equipment failure rate.

### 2.5 Electrolyzer model

The anode and cathode of the electrochemical cells are saturated and elastically distorted by water vapor in this model. In the electrochemical cells, the pressure and temperature in the gas flow canals were considered to remain constant. Water vapor enthalpy was also considered to remain constant throughout the operating temperature range. The model considers the electrochemical cells' operating temperature but ignores the energy required for hydrogen compression and water delivery. Furthermore, parasitic losses hinder the system from attaining its full capability.

The rate of hydrogen production can be achieved by the following [21] [Eq. (5)]:

(5) $$r_{hp}=\frac{\eta_{I}\times I_{el}\times N_{el}}{2\times F}$$

where, $I_{el}$, $N_{el}$, and F represent, in turn, the current density of the Electrolyzer, the number of the electrolytic cells, and the Faraday constant. The standard value of the Faraday constant is F=96487C/mol.

According to the Faraday Law, the experimental hydrogen rate can be achieved [Eq. (6)]:

(6) $$\eta_{c}=96.5\times \exp\left(\frac{0.09}{I_{el}}-\frac{75.5}{I_{el}^{2}}\right)$$

where, $\eta_{cur}$ represents the current efficacy of the Electrolyzer.

The power output of an Electrolyzer can be determined using the following formula [Eq. (7)]:

(7) $$P_{EL}^{Tank}=P_{RE}^{EL}\times \eta_{EL}$$

where, $P_{RE}^{EL}$, and $\eta_{EL}$ represent, in turn, the applied power into the Electrolyzer by renewable units, and the Electrolyzer efficiency.

The efficiency of an Electrolyzer is determined by factors, such as its design, utilized materials, and operating circumstances. Higher efficiency leads to more hydrogen generated for the same quantity of electrical power input.

The quantity of electrical power input and the electrolysis effectiveness define the current flowing through the Electrolyzer. The working parameters, including electrolyte concentration and temperature determine the voltage across the Electrolyzer.

Power loss in an Electrolyzer can occur owing to electrical circuit impedance, the heat created by the electrolysis process, and other variables. Minimizing power loss is critical for optimizing the Electrolyzer's energy efficiency and hydrogen generation.

### 2.6 Fuel cell

Understanding and enhancing the efficiency of a Proton Exchange Membrane Fuel Cell (PEMFC) stack requires a mathematical model for the total output voltage. The model is developed by considering numerous elements that influence the voltage output of the fuel cell stack. The PEMFC stack is made up of several separate fuel cells, all with a cathode, an anode, and a Proton Exchange Membrane. The anode and cathode are usually formed of a catalyst-coated layer of carbon material, whereas the proton exchange membrane is constructed of a polymer. Through an electrochemical mechanism, the fuel cell stack turns the chemical energy of oxygen and hydrogen into electrical energy.

The mathematical model of the total output voltage of the PEMFC stack takes various elements into account, including operating circumstances, such as fuel cell pressure and temperature, reactant gas flow rate, and fuel and oxidant composition. The model also takes into account the fuel cell stack's internal resistance, which might alter the voltage output. By summing the individual voltage outputs of each fuel cell in the stack, the overall output voltage of the PEMFC stack is computed. The electrochemical processes occurring at the anode and cathode, as well as the electrical conductivity of the PEM, influence the voltage output of each fuel cell. The model also considers the consequences of a mass movement, including the diffusion of reactant gases and products of the reaction. The model also considers the consequences of a mass movement, including the diffusion of reactant gases and products of the reaction [Eq. (8)].

(8) $$V_{o}=N_{nc}\times \left(E_{oc}-E_{op}-E_{O}-E_{ops}\right)$$

where, $N_{nc}$, $E_{oc}$, $E_{op}$, $E_{O}$, and $E_{ops}$ represent, in turn, the number of connected cells, the open circuit potential condition per cell, the activation over-potential of the cells, the Ohmic voltage drop in the cells, and the over-potential saturation in each cell [(Eqs. (9), (10), (11)].

To estimate the PEMFC's so-called reversible voltage open circuit voltage, the following information which includes temperatures below 100 °C is defined [Eqs. (12) and (13)]:

(9) $$E_{oc}=1.23-8.5\times 10^{-4}\left(T_{FC}-298.15\right)+4.31\times 10^{-5}{\times T}_{FC}\times \ln\left(P_{H_{2}}\sqrt{P_{O_{2}}}\right)$$

where,

(10) $$P_{O_{2}}=R_{hc}\times P_{H_{2}O}\left[\frac{1}{\frac{R_{hc}\times P_{H_{2}O}}{P_{c}}\times e^{\frac{1.635I_{FC}/A}{T^{1.334}I_{FC}}}}-1\right]$$

(11) $$P_{H_{2}}=\frac{R_{ha}\times P_{H_{2}O}}{2}\left[\frac{1}{\frac{R_{ha}\times P_{H_{2}O}}{P_{a}}\times e^{\frac{1.635I_{FC}/A}{T^{1.334}I_{FC}}}}-1\right]$$

(12) $$\log_{10}\left(P_{H_{2}O}\right)=2.95\times 10^{-2}T_{c}-9.18{\times 10}^{-5}T_{c}^{2}+1.4{\times 10}^{-7}T_{c}^{3}-2.18$$

(13) $$T_{c}=T_{FC}-273.15$$

Here, 25 °C is taken into consideration as the reference temperature value during operational fluctuations.

Equation (14) provide the mathematical model of the activation over-potential:

(14) $$E_{op}=-\left[\gamma_{1}+\gamma_{2}T_{FC}+\gamma_{3}T_{FC}\;\ln\left(C_{O_{2}}\right)+\gamma_{4}T_{FC}\;\ln\left(I_{FC}\right)\right]$$

The oxygen saturation in the cathode's catalytic interface, denoted by the symbol $C_{O_{2}}$, is calculated as follows [Eq. (15)]:

(15) $$C_{O_{2}}=\frac{P_{O_{2}}}{5.1\times 10^{6}}\times e^{\frac{498}{T_{FC}}}$$

The cathode's catalytic interface has a hydrogen saturation (mol/cm3) as follows [Eq. (16)]:

(16) $$C_{H_{2}}=\frac{P_{H_{2}}}{1.1\times 10^{6}}\times e^{\frac{-77}{T_{PEM}}}$$

The Ohmic voltage loss can be mathematically defined by equation (17), equation (18) and equation (19):

(17) $$E_{O}=\left(R_{m}+R_{c}\right)\times I_{FC}$$

where,

(18) $$R_{m}=\rho_{m}l/S$$

(19) $$\rho_{m}=\frac{181.6\left[0.062{\left(\frac{T_{PEM}}{303}\right)}^{2}{\left(\frac{I_{PEM}}{S}\right)}^{2.5}+0.03\left(\frac{I_{PEM}}{S}\right)+1\right]}{\left[\lambda -0.063-3\left(\frac{I_{PEM}}{S}\right)\right]\times e^{\frac{T_{PEM}-303}{T_{PEM}}}}$$

Finally, the saturation loss voltage ($E_{ops}$) can be mathematically defined by Eq. (20):

(20) $$E_{ops}=-\beta \;\ln\left(1-\frac{J}{J_{\max}}\right)$$

where, I signifies the membrane thickness, $I_{FC}$ specifies the operating current for the fuel cell, S defines the membrane surface ($cm^{2}$), λ defines an adjustable parameter, $T_{fc}$ describes the fuel cell temperature, $\beta_{i}$ designates the experimental coefficients, $\rho_{m}$ signifies the resistivity of the membrane, β represents a parametric coefficient, J represents the density of the real current, and $J_{\max}$ specifies the maximum value of J. $CH_{2}$ and $CO_{2}$ are the saturation of the hydrogen and oxygen, in addition, $P_{H}$, $P_{O_{2}}$, and $P_{H_{2}O}$ represent, in turn, the partial pressure for hydrogen, oxygen, and the water. Finally, $R_{ha}$ and $R_{hc}$ describe, in turn, the vapor relative humidity at anode and cathode, Pc and Pa describe, in turn, the inlet pressure for the cathode and the anode. The parameter values, in this study, are achieved based on [22] and Table [1].

**Table 1:** Model's parameters.

$\beta_{1}$	$\beta_{2}$	$\beta_{3}$	$\beta_{4}$	β	λ	$R_{c}$
−1.03	3.35e-3	7.24e-5	−0.95	1.36e-2	10	1e-4

### 2.7 Inverter model

Direct current (DC) power, which is generated by solar panels and battery energy storage devices, is incompatible with the AC (Alternating Current) electricity used by the majority of homes and businesses. An inverter is required to transform the DC power into AC electricity so that it may be used.

An electrical equipment, known as an inverter, transforms DC electricity generated by solar panels or battery storage systems into AC electricity that may be used to power appliances and other electrical devices. The inverter achieves this by converting the DC voltage into a sine wave with the same frequency and voltage as the AC power grid using a number of electrical components, including capacitors and transistors. The following formula [Eq. (21)] may be used to determine the power output of the inverter:

(21) $$P_{inv}=\left[P_{FC}^{inv}+P_{RE}^{inv}\right]\times \eta_{inv}$$

where, $P_{h}^{D}$ describes the hourly demand and $\eta^{Inv}$ specifies the inverter efficiency.

## 3 Reliability indicators

The efficiency of power systems and their capacity to satisfy the demands of the load are both evaluated using reliability indices, which are vital instruments. These indices offer a numerical assessment of the supply's dependability and likelihood of breakdowns or interruptions. The load interruption hope index, a gauge of the anticipated number of interruptions a load will encounter during a specific time period, is one such indication.

Power system managers and engineers may assess the system's efficiency and pinpoint opportunities for development by employing reliability indicators. The system may be having numerous and lengthy interruptions, for instance, if the load interruption hope index is high. This issue might be resolved by making changes to the system's design, maintenance, or operation. The definition of the load interruption hope index is given below [Eq. (22)]:

(22) $$LOLE=\sum_{t=1}^{M}E\left[L\left(t\right)\right]$$

The mathematical expectation of load loss in the time step t, denoted by E[L(t)], is calculated using the following relation [Eq. (23)].

(23) $$E\left[L\left(t\right)\right]=\sum_{s\in S}T_{s}\times P_{s}$$

The equation above defines the calculation of the energy loss expectation index, where $P_{s}$ represents the system probability in situation s, S signifies the set of all possible situations for the system, and $T_{s}$ represents the duration of the load interruption if the system is in situation s. In simpler terms, the energy loss expectation index is computed by the use of Eq. (24).

(24) $$EENS=\sum_{t=1}^{M}E\left[LOE\left(t\right)\right]$$

where, E[LOE(t)] specifies lost load expectation at time t and is achieved by Eq. (25):

(25) $$E\left[LOE\left(t\right)\right]=\sum_{s\in S}Q_{s}\times P_{s}$$

where, $Q_{s}$ specifies the load lost value at position s.

Afterward, the LPSP is achieved as follows [Eq. (26)]:

(26) $$LPSP=\frac{LOEE}{\sum_{t=1}^{M}D\left(t\right)}$$

In the equation mentioned above, D(t) is the measured load demand quantity at time step t. Additionally, the cut-off factor, which is equivalent to the load, can be expressed using the following formula as well [Eq. (27)].

(27) $$ELF=\frac{1}{M}\sum_{t=1}^{M}\frac{Q\left(t\right)}{D\left(t\right)}$$

The objective of this study is to determine the ideal capacity of the decision-making variables that comprise the system equipment, such as the number and orientation of Solar Arrays, quantity of Wind Turbines, capability of the Fuel Cell, Electrolyzer, Hydrogen Storage Unit, and Inverter. This will be achieved by minimizing the energy production costs of the system through the application of a modified metaheuristic algorithm, which has been known as Improved Al-Biruni-Algorithm. The subsequent section will provide an explanation of this optimizer.

## 4 Objective function

Combined system equipment net cost refers to the total cost of purchasing and installing all necessary components of the system. These costs include the costs of acquiring or leasing hardware, software, and other related equipment, as well as installation, configuration, and maintenance costs. The Net Present Cost of combined system equipment can be determined by subtracting any applicable discounts, rebates, or subsidies from the total cost of acquisition and installation.

Accurately determining the net cost of combined system equipment is critical as it affects the total budget and feasibility of any project or initiative. A clear understanding of net costs enables organizations to make informed decisions about the equipment and systems to invest in and how to allocate resources for optimal efficiency and effectiveness. The net present cost of combined system equipment can be calculated as follows [Eq. (28)]:

(28) $$NPC_{j}=N_{j}\times \left(C_{j}^{R}\times K{+C}_{j}^{O\&M}\times PWA+C_{j}^{C}\right)$$

where, N defines the capacitance of the equipment or units' quantity, K signifies the fixed payments' current cost factor, PWA represents the current value factor of annual costs, and $C^{C}$, $C^{R}$, and $C^{O\&M}$ represent, in turn, the cost of primary investment, the substitute cost, and the annual repair as well as maintenance cost. The current value factor of annual cost and the fixed payments' current cost factor can be achieved as follows [Eqs. (29), (30), (31)]:

(29) $$PWA=\frac{{\left(1+\alpha \right)}^{R}-1}{\alpha {\left(1+\alpha \right)}^{R}}$$

(30) $$K_{j}=\sum_{n=1}^{y_{j}}\frac{1}{{\left(1+\alpha \right)}^{n.L_{j}}}$$

where,

(31) $$ir=\frac{\alpha_{n}-i}{1+i}$$

Here, i describes the inflation rate, R describes the duration of the project during 20 years, α represents the actual interest rate (here, 6%), L signifies the replacement costs of the corresponding equipment, and $\alpha_{n}$ specifies the nominal interest rate. The Net Present Cost for the loss of load can be achieved by Eq. (32):

(32) $$NPC_{loss}=LOEE\times C_{l}\times PWA$$

where, $C_{l}$ specifies the average loss value formed by the interruption of the consumption kilowatt hours. Eq. (33) describes the considered objective function that should be optimized.

(33) $$Obj=\min_{x}\left(\sum_{i}NPC_{i}+NPC_{loss}\right)$$

where, i describes the desired equipment, and x describes the number of decision variables. The constraints of the objective function are as follows [Eqs. (34), (35), (36)]:

(34) $$0\leq \theta_{PV}\leq \frac{\pi }{2}$$

(35) $$E\left(ELF\right)\leq ELF_{\max}$$

(36) $$E_{Tank}\left(0\right)\leq E_{Tank}\left(8760\right)$$

where, $\theta_{PV}$ represents the installation angle of the PV system.

## 5 Modified Al-Biruni earth radius (MBER) algorithm

### 5.1 Algorithm background

One of the Al-Biruni's astronomical works was determination of the Earth radius in the 11th century. The space amid the horizontal axis and ground from top of a hill was measured for this purpose. Al-Biruni determined this distance two times. First, he measured the height of a hill (h). Then, he determined the radius of the hilltop from 2 diverse points ($\varphi_{1}\;and\;\varphi_{2})$. Based on Eq. (37), the height of the hill could be calculated.

(37) $$h=\frac{d\;\tan\;\varphi_{1}\;\tan\;\varphi_{2}}{\tan\;\varphi_{2}-\tan\;\varphi_{1}}$$

At the second step, Al-Biruni gaged the peak of mountain and determined the dip of the horizon. Using this date, the Earth radius could be calculated as below [Eq. (38)]:

(38) $$R_{Earth}=\frac{hcos\beta }{1-\cos\;\beta }$$

Based on the Al-Biruni procedure, an algorithm is offered for the cooperative optimizer's exploitation and exploration, which models the population for attaining a global optimization. In nature, populations or swarms get together and collaborate to accomplish their aims. Colonies ants and bees might be an instance of such collaborations in which every individual of the population makes a distinctive influence in the swarm. Some workers of the swarm go out from the group for food gathering. The motivation of the offered optimizer is that the individuals of the group have been divided into sub-sets to accomplish numerous actions at diverse periods and cooperate for their aims. The exploitation and exploration phases are generally executed to discover the finest result for optimizing the problem. In this paper, the A-BERO does split members into two sets, and every individual is responsible for some actions. In the proposed optimizer, the phases of exploration and exploitation guarantee the investigation of the solution area to avoid the local optimal occurrence. The vast array of population-based optimizers requires each member to accept exploitation after epochs, which might lead to the avoidance of local optima. It is done by keeping a set of individuals with whom more regions have been searched over the solution space. What is more, the offered optimizer rapidly enhances the agents who search the solution area, providing that there is not any improvement in the performance of the method after three repetitions over the search area.

### 5.2 Basic conceptions and modeling

The main aim of the offered optimizer is to gain an optimum result with some limitations for an issue. In this optimizer, a vector is utilized for representing each member in the swarm [Eq. (39)].

(39) $$\vec{A}=\{A_{1},A_{2},\ldots ,A_{c}\}\epsilon R_{c}$$

Here, $A_{c}$ signifies a parameter in the optimizing process, and the size of solution area is represented by c. A cost function (f) has been utilized in the offered procedure to determine the quality of the performance of a member to a specified point. The method of optimizing utilizes the subsequent stages for exploration over populations for a particular optimal vector $\vec{A}$ that makes the cost function maximum. The optimizing procedure commences with a set of random agents (results). To commence the optimizing method, the next parameters are required: the cost function, the lowest and highest boundaries for every solution, the size of population, and the dimension.

### 5.3 Balance of exploitation-exploration

In the offered method, when the population has been split into subgroups, the members' quantity in each set has been dynamically altered to improve the balance among exploitation and exploration phases. The commencement of the method is to split the population into two sets for exploitation and exploration. Seventy percent of the population is the dimension of the exploration set, while the dimension exploitation set is thirty percent of the individuals in a population. For modifying the cost function amount of agents in each set, the agents' quantity in the phase of exploitation has been set at thirty percent at initial, and then it increases throughout the epochs to seventy percent of the agents' number. However, the agents' number in the exploration set decreases to thirty percent over epochs, whereby the primary number has been set to seventy percent. This procedure permits more and more significant modification in the cost value of agents’ global mean. What is more, the elitism scheme has been utilized to ensure the optimizing method convergence. Elitism is applied by keeping the leading result of the process when there is no finer result. In the offered approach, when there is not a significant improvement in the cost value of a result for three epochs, the local optimal result could gain. Subsequently, another exploration agent could be produced using the operation of mutation.

### 5.4 Exploration phase

The exploration stage is responsible for finding existing situations over the solution area. It is also responsible for avoidance of local optimal when the method is moving towards the finest result as expressed in the following part.

### 5.5 Moving towards the finest result

The agents of the exploration set utilize this approach for seeking potential areas around its existing situation in the solution area. It has been obtained via searching constantly amid adjacent possible alternatives for a better amount of cost function. The [Eqs. (40) and (41)] below formulas have been used for this purpose:

(40) $$\vec{D}=\vec{r_{1}}.\left(\vec{A}\left(t\right)-1\right)$$

(41) $$\vec{A}\left(t+1\right)=\vec{A}\left(t\right)+\vec{D}.\left(2\vec{r_{2}}-1\right)$$

where, the result vector at the *t*th result is represented by $\vec{A}\left(t\right)$, and $\vec{D}$ is the circle diameter, whereby the search individual would search promising zones. $\vec{r_{1}}$ and $\vec{r_{2}}$ represent the vectors of the coefficient, which can be determined based on the formula below [Eq. (42)]:

(42) $$r=\frac{b\;\cos\left(y\right)}{1-\cos\;\left(y\right)}$$

Here, b represents a randomly chosen amount between zero and two, and yϵ(0,180].

### 5.6 Exploitation phase

Improvement of the present result is the responsibility of the exploitation set. The offered algorithm determines the cost amount of each member over each epoch and separates the finest member. 2 diverse processes are utilized for gaining exploitation, as expressed in the next sections.

### 5.7 Moving towards the finest result

For moving the search individuals towards the finest result, the [Eqs. (43) and (44)] below formulas have been utilized:

(43) $$\vec{A}\left(t+1\right)=r^{2}\left(\vec{D}+\vec{A}\left(t\right)\right)$$

(44) $$\vec{D}=\vec{r_{3}}\left(\vec{K}\left(t\right)-\vec{A}\left(t\right)\right)$$

Here, $\vec{r_{3}}$ signifies a random vector, which is determined like $\vec{r_{1}}$ and $\vec{r_{2}}\text{.}$ This vector does control the steps of motion towards the finest result. $\vec{A}\left(t\right)$ represents the vector of result at the $t^{th}$ epoch, $\vec{D}$ illustrates the vector of distance, and $\vec{K}\left(t\right)$ demonstrates the vector of the finest result.

### 5.8 Investigating area adjacent the finest result

The adjacent area of the finest result (leader) is the most promising one. Accordingly, a number of members search the neighboring of the finest result with the ability of discovering far finer result. The [Eqs. (45) and (46)] below formulas have been used in this operation.

(45) $$\vec{A}\left(t+1\right)=r\left(\vec{l}+\vec{A^{*}}\left(t\right)\right)$$

(46) $$\vec{l}=x+\frac{2t^{2}}{{t_{tot}}^{2}}$$

Here, the finest result is illustrated by $\vec{A^{*}}\left(t\right)$, x signifies a random amount between zero and one, t and $t_{tot}$ demonstrate, in turn, the epoch number, and the total quantity of epochs.

### 5.9 Operation of mutation

Another approach that is utilized by the offered algorithm is mutation. It has been a genetic operative employed for creating and sustaining the variety of population groups. It might be assumed as a feasible local random distraction for members of the population that leads to the avoidance of premature convergence via preventing local optimum like a move in the functions of solution area to another best case. Actually, for the finest potential of exploration in the offered optimizer, the mutation has been vital and significant [Eq. (47)].

(47) $$\vec{A}\left(t+1\right)=x^{2}*\vec{l}-\frac{b\;\cos\;\left(y\right)}{1-\cos\;\left(y\right)}$$

### 5.10 Modified Al-Biruni earth radius (BER) algorithm

This part describes an improved version of the Al-Biruni-Algorithm, using Chaos Map and Lévy Flight (LF). The LF is used to create a more distributed primary swarm. However, chaos maps help you find a balance between stages of exploration and exploitation. Such improvements are used to improve convergence speed and fix local optimal.

#### 5.10.1 The Lévy Flight mechanism

Various approaches have been presented to address a method with better efficiency. Among them, the Lévy flight (LF) mechanism [23] is a commonly utilized technique for enhancing the effectiveness of meta-heuristics. The LF mechanism operates by utilizing random walk behavior to control the local search position, as stated below [Eqs. (48), (49), (50)]:

(48) $$Lf\left(w\right)\approx w^{-1}\times w^{-\tau }$$

(49) $$w=A{\times \left|B\right|}^{-1/\tau }$$

(50) $$\sigma^{2}={\left\{\frac{\sin\;\left(\pi \tau /2\right)}{2^{\left(1+\tau \right)/2}}\times \frac{\Gamma \left(1+\tau \right)}{\tau \Gamma \left(\left(1+\tau \right)/2\right)}\right\}}^{\frac{2}{\tau }}$$

where, w indicates step size, Γ(.) specifies a Gamma function, and $A,B∼N\left(0,\sigma^{2}\right)$. By assuming τ=1.5 [24], the heading to the best solution can be achieved as follows [Eq. (51)]:

(51) $$S\left(t+1\right)=S\left(t\right)+D\times \left(2r_{2}-1\right)\times Lf\left(\sigma \right)$$

#### 5.10.2 Chaos map

Instead of using random parameters, this method employs chaotic parameters with unpredictable behavior. Chaos sequences are non-repetitive and divergent patterns observed in nonlinear and dynamic systems. As a result, this approach results in more efficient convergence for simple searches than other probable-based methods [25]. The Al-Biruni-Algorithm utilizes chaotic variables, rather than random ones, to achieve better exploration of the solution space due to the dynamic nature of the turbulence sequence [26].

There are various forms of chaotic maps employed in optimizers, and by modifying the initial conditions, different sequences can be generated with ease. In this study, the sinusoidal map is utilized to facilitate faster convergence of the Al-Biruni-Algorithm and to achieve a balance between exploration and exploitation for better solutions to avoide local optimal. To enhance the Al-Biruni-Algorithm using the sinusoidal map, the random values in the chaos function are replaced with pre-determined values. The mathematical expression for the sinusoidal map is given in the following [Eq. (52)]:

(52) $$z_{k+1}^{new}=a{\left(z_{k}^{new}\right)}^{2}\sin\left(\pi z_{k}^{new}\right)\text{,}$$

Here, $z^{t}$ describes a chaotic random value for the iteration t, a is a constant number in the range (0,4], and k defines the control parameter equal to 2.3. Here, $z^{0}=0.5$.

### 5.11 Algorithm authentication

A series of test functions are used to validate the efficiency of the suggested Modified Al-Biruni-Algorithm in order to evaluate its effectiveness. The first ten benchmark functions from the “CEC-BC-2017 test suite,” which are often used to gauge the performance of optimization methods, were the main subject of the study. To provide a fair comparison with other algorithms, the decision variable bounds for the test functions have been set between −100 and 100. This method made it easier to evaluate the suggested algorithm consistently and uniformly so that it could be contrasted with other tested and refined algorithms.

The proposed Modified Al-Biruni-Algorithm has been compared to a total of four optimization approaches, including the Tunicate Swarm Algorithm (TSA) [27], Sine Cosine Algorithm (SCA) [28], Gravitational Search Algorithm (GSA) [29], and the original Al-Biruni Earth Radius (BER) [30]. These algorithms have been chosen due to their widespread adoption in the literature and their success in solving optimization problems. The comparison's goal is to assess the effectiveness of the suggested Modified Al-Biruni-Algorithm and to pinpoint its benefits and drawbacks with regard to other cutting-edge algorithms. The parameter value for the examined methods is shown in Table 2.

**Table 2:** Value of a parameter for the examined algorithms.

Algorithm	Parameter	Value
TSA [27]	Search agents	200
	$P_{\min}$	1
	$P_{\max}$	4
	Number of generations	1000
SCA [28]	Search agents	200
	Number of elites	2
	Number of generations	1000
GSA [29]	Search agents	200
	Gravitational constant	100
	Alpha coefficient	20
	Number of generations	1000

Optimization methods might not always produce a globally optimum solution because of the algorithm's random initialization. They are able to swiftly locate a poor answer, albeit, that is extremely near to the ideal one. Therefore, 25 simulations of each of the functions were performed. Essential metrics like the average value (Avg) and standard deviation (StD) values might be more easily analyzed as a result. The average number offers the mean outcomes for the 30 runs, whilst the standard deviation aids to assess the findings' volatility. The Modified Al-Biruni Earth Radius (BER) Algorithm is numerically compared to other approaches in Table 3.

**Table 3:** Modified Al-Biruni Earth Radius (MBER) Algorithm compared with other approaches.

Benchmark		MBER	BER [30]	GSA [29]	SCA [28]	TSA [27]
F1	Avg	0.00	3.45	4.68	5.63	3.26
	StD	0.00	2.96	3.49	4.51	2.35
F2	Avg	2.15	4.85	5.78	4.36	3.86
	StD	1.25	3.62	4.39	3.45	3.25
F3	Avg	0.00	4.5e-10	2.56e-8	5.36e-9	3.85e-8
	StD	0.00	0.02	2.95e-6	5.51e-4	3.72e-3
F4	Avg	0.00	0.00	4.24e-8	7.24e-7	6.91e-8
	StD	0.02	0.03	3.72e-7	6.85e-7	5.82e-8
F5	Avg	0.00	3.21	4.34	6.67	3.36
	StD	0.00	2.69	3.67	5.83	3.13
F6	Avg	0.05	6.36	7.49	7.13	5.69
	StD	0.00	5.49	6.37	6.78	4.93
F7	Avg	0.00	6.24	5.63	6.83	6.45
	StD	0.72	5.46	4.51	5.32	5.72
F8	Avg	0.00	0.07	0.97	1.71	2.13
	StD	0.00	0.01	0.62	1.19	1.05
F9	Avg	0.00	0.00	0.63	1.26	1.82
	StD	0.00	0.00	0.54	1.13	1.73
F10	Avg	0.00	0.49	3.81	5.34	3.84
	StD	0.00	0.36	3.25	4.57	2.39

As can be concluded from the results, for benchmark function F1, the MBER Algorithm achieved an average value of 0.00, outperforming all other algorithms, including BER, GSA, SCA, and TSA. The standard deviation (StD) for this function was also low, indicating consistent performance across simulations. For benchmark function F2, the MBER Algorithm obtained an average value of 0.25, which was slightly higher than the average values achieved by the SCA and GSA algorithms. However, the difference was relatively small, suggesting a competitive performance. The StD for this function was moderate compared to other algorithms. In the case of benchmark function F3, the MBER Algorithm once again exhibited a strong performance with an average value of 0.00, surpassing all other algorithms. The StD for this function was also relatively low, indicating consistent results. For benchmark functions F4 and F5, the MBER Algorithm achieved average values of 0.00, showcasing its effectiveness in finding optimal or near-optimal solutions. The StD values for these functions were also low, indicating consistent performance. In the case of benchmark function F6, the MBER Algorithm obtained an average value of 0.58, which was higher compared to the TSA algorithm but lower than the values achieved by BER, GSA, and SCA. The StD for this function was moderate compared to other algorithms. For benchmark function F7, the MBER Algorithm once again demonstrated its superiority with an average value of 0.00, outperforming all other algorithms. The StD for this function was low, indicating consistent performance. In the case of benchmark function F8, the MBER Algorithm achieved an average value of 0.00, showcasing its effectiveness in finding optimal or near-optimal solutions. However, the StD for this function was slightly higher compared to the TSA algorithm. For benchmark functions F9 and F10, the MBER Algorithm achieved average values of 0.00 and 0.26, respectively. While it exhibited a competitive performance for F9, it could outperform algorithms, including GSA, SCA, and TSA; however, its value was equal to BER.

## 6 Results and discussions

### 6.1 Case study

According to the findings provided by experts hailing from prestigious institutions, such as Harvard, Tsinghua, Nankai, and Renmin Universities of China, it has been determined that solar energy possesses the capacity to fulfill 43.2% of China's electricity requirements by the year 2060, while maintaining a justifiable cost [31]. The fact that this conclusion suggests that China has the ability to dramatically enhance its renewable energy capacity in the future, even though it is not directly connected to discovering small cities in China with high potential for energy production by Solar Panels, Wind Turbines, and Fuel Cells.

There are a few things to take into account when picking certain small cities in China that have a high potential for energy generation using fuel cells, solar panels, and wind turbines. First and foremost, it is critical to seek towns with plenty of natural resources for generating energy, such as lots of sunshine or wind. The policies and incentives that have been put in place by the local government to promote the development of renewable energy sources may also have an impact on the amount of energy that can be generated in a certain city. Dunhuang is one of the areas that has a significant potential based on [31]. The geographical coordinates for this city are 40°08′28″N 94°39′50″E.

**Fig. 2:**
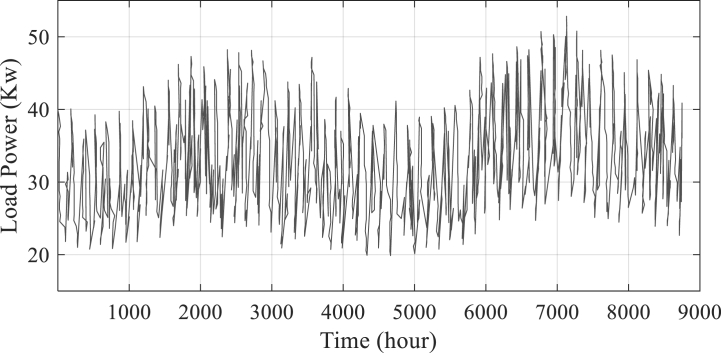
Time-based load profile for the investigated scenario.

Dunhuang, which lies in the northwest Chinese province of Gansu, is renowned for having abundant sunlight. With a concentration on solar and wind energy, Dunhuang has shown tremendous potential for energy generation, using fuel cells, solar panels, and wind turbines. The city is a desirable location for renewable energy investment and a possible role model for sustainable development in China due to its wealth of natural resources, supporting government policies, and current facilities for the growth of renewable energy sources. The load profile based on power consumption (kW) for the investigated situation is displayed in Fig. 2.

**Fig. 3:**
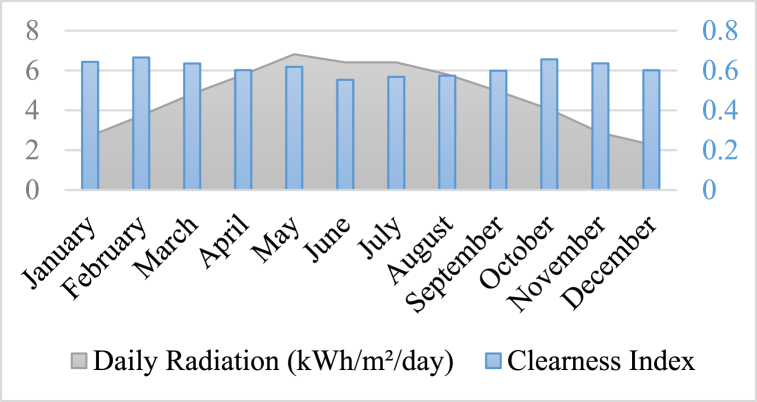
The meteorological patterns at the study site, including the clearness index and daily radiation.

**Fig. 4:**
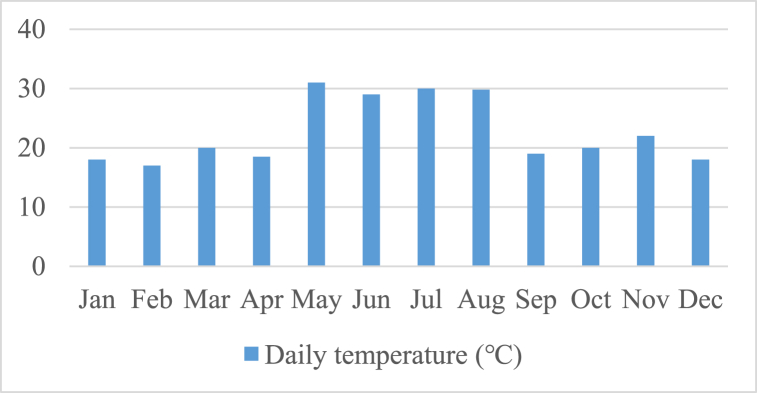
The meteorological patterns at the study site, including the average daily temperature.

**Fig. 5:**
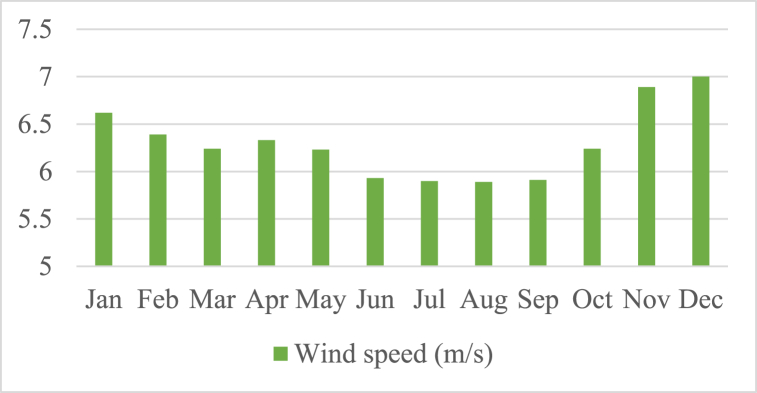
The meteorological patterns at the study site, including the wind speed.

In order to meet the load requirements when the Photovoltaic (PV) panels are unable to provide sufficient energy, a storage tank unit is integrated into the system. Based on meteorological data collected from the National Aeronautics and Space Administration (NASA) [32], an assessment is conducted to evaluate the effectiveness of the method. The load demand exhibited minimum and maximum values of 19.92 kW and 52.82 kW, respectively. In Fig. 3, Fig. 4, Fig. 5, the meteorological patterns at the study site, including the clearness index, daily radiation, the wind speed, and temperature are depicted. These figures provide a comprehensive understanding of the weather conditions at the site, which are critical factors in determining the optimal design and operation of the system. Table 3 presented herein provides information on the Clearness Index and Daily Radiation for each month of the year. The Clearness Index is a measure of the ratio of solar radiation received at a specific location to the solar radiation that would be received if the sky were entirely clear. A higher Clearness Index indicates a greater proportion of solar radiation reaching the ground. The Daily Radiation column, expressed in kilowatt-hours per square meter per day, represents the average amount of solar radiation received daily.

Upon analyzing the data, it is evident that February (0.665) and October (0.656) record the highest Clearness Index values, indicating relatively clear skies during these months. Conversely, June (0.553) and July (0.568) exhibit the lowest Clearness Index values, suggesting that these months experience more cloud cover and potentially reduced solar radiation. The peak values for Daily Radiation are observed in May (6.815 kWh/m^2^/day) and April (5.787 kWh/m^2^/day). These months likely receive the highest amount of solar radiation, contributing to increased energy potential. Conversely, December (2.279 kWh/m^2^/day) and November (2.874 kWh/m^2^/day) exhibit the lowest Daily Radiation values, indicating lower solar radiation levels during these winter months.

Upon analysis of the data, it is evident that certain trends can be observed. The months of May, June, July, and August exhibit the highest average daily temperatures, with readings of 31 °C, 29 °C, 30 °C, and 29.8 °C, respectively. These months are typically associated with the summer season and are characterized by warmer weather. Conversely, the winter months of January, February, and December display the lowest average daily temperatures, with readings of 18 °C, 17 °C, and 18 °C, respectively. These months are generally colder as they fall within the winter season. The months of March, April, September, October, and November exhibit moderate average daily temperatures, with readings ranging from 18.5 °C to 22 °C. These months often serve as transitional periods between seasons and may experience varied weather conditions.

It is important to note that the aforementioned temperatures are average daily readings and may vary from day to day. Furthermore, this data provides a general overview and does not account for specific regional or local variations. Weather patterns can be influenced by various factors, such as geographical location, altitude, proximity to water bodies, and climate zones.

To obtain a comprehensive understanding of the weather conditions for a specific location, it is advisable to consult localized weather reports or historical climate data that provide a more detailed analysis of temperature variations throughout the year.

Upon analysis of the data, it is evident that certain trends can be observed. The months of December, November, and January exhibit the highest average of wind speeds, with values of 7 m/s, 6.89 m/s, and 6.62 m/s, respectively. These months are characterized by stronger winds, which may be attributed to weather systems associated with winter and transitional periods.

Conversely, the months of June, July, August, and September exhibit relatively lower average wind speeds, with values of 5.93 m/s, 5.9 m/s, 5.89 m/s, and 5.91 m/s, respectively. These months fall within the summer season when atmospheric conditions may be more stable and less prone to strong winds.

The remaining months from February to May and October exhibit moderate average wind speeds ranging from 6.23 m/s to 6.33 m/s. These months typically experience intermediate wind speeds, suggesting a mix of calm and slightly breezy conditions.

It is important to note that these average wind speeds may vary throughout the day and across different locations. Local topography, proximity to water bodies, and other geographical factors can influence wind patterns and intensity.

For a comprehensive understanding of wind conditions in a specific area or for precise applications, such as wind energy generation or outdoor activities, it is advisable to consult detailed regional or local wind data, including prevailing wind directions and gust strengths, which can provide more accurate insights.

In Table 4, you can find a comprehensive overview of the parameter values associated with the hybrid system equipment that is analyzed in this study.

**Table 4:** Values of the hybrid system equipment's parameters.

Tools	The cost of initial investment (US$/unit)	The cost of Replacement (US$/unit)	The cost of yearly repair and maintaining (US$/unit-yr)	Valuable life of equipment (years)	efficiency (percentage)	Availability (percentage)
Fuel cell	4500	2800	220	15	70	100
Solar array	9000	8000	40	40	–	98
Electrolyzer	4000	1850	45	40	90	100
Wind Turbine	22,630	17,500	90	40	–	98
Hydrogen tank	1550	1460	40	40	107	100
inverter	950	900	28	30	103	99.85

The table comprehensively analyzes various equipment types in a hybrid system, including fuel cells, solar arrays, Electrolyzers, wind turbines, hydrogen tanks, and inverters. It outlines the initial investment required for each type, including the cost of replacement, the annual cost of repair and maintenance, the valuable life of each equipment type, the efficiency of each equipment type, and the availability of each equipment type. The cost of a single fuel cell is $4500, while the cost of replacing a solar array is $8000 per unit. The annual cost of repairing and maintaining each equipment type is $45 per unit each year. The efficiency of each equipment type is 70%, and the availability of each equipment type is expressed as a percentage. The table provides valuable insights into the cost, performance, and reliability characteristics of different hybrid system equipment types, aiding in analyzing and comparing their implications within the hybrid system. Table 5, on the other hand, presents the specific conditions that were assumed when evaluating the performance of the system.

**Table 5:** Conditions of the case study.

useful life of the system	Real interest rate	${ELF}_{\max}$	Load pattern	Load peak	Cost of load loss
40 years	9%	0.04	IEEE RTS	70 KW	7.3 US$/KWh

The aforementioned table comprises parameters that are essential for the evaluation of a hybrid system. The system's useful life is estimated to be 40 years, and the real interest rate is set to 9%. The maximum energy loss fraction has been determined to be 0.04, with the objective of limiting losses to 4% or less. The load pattern adheres to the IEEE RTS standard, with the highest level of demand that is 70 kW. The cost of load loss has been calculated to be 7.3 US dollars per kilowatt-hour (kWh). These parameters serve to provide a contextual background for the case study and establish a framework for the evaluation of the hybrid system. The load peak represents the highest level of electricity demand within the load pattern.

### 6.2 Simulation results

**Fig. 6:**
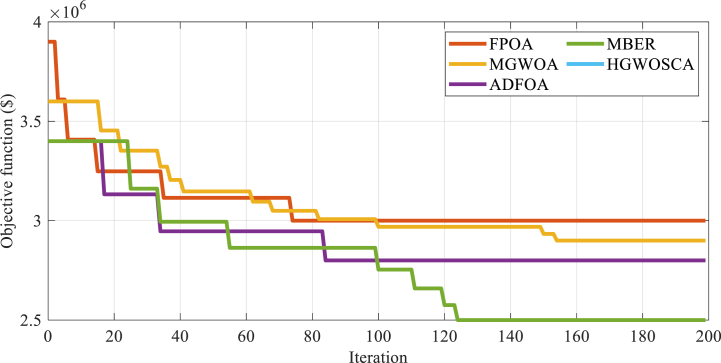
Convergence analysis of the optimizers used to solve the problem.

This section presents the results obtained from a hybrid system that was optimized using the Modified Al-Biruni Earth Radius (MBER) Algorithm. The algorithm was repeated 50 times to ensure accurate and reliable results. The optimization method considered several decision-making variables, including the number of PV arrays, the number of Wind Turbines, the capacity of the hydrogen tank, the inverter and Fuel Cell power, the power of the Electrolyzer, and the angle of the solar array relative to the sun's radiation. In order to provide a comprehensive analysis, the results obtained from this optimization technique were compared with those obtained from four other optimization techniques: Amended Dragon Fly Optimization Algorithm (ADFOA), Modified-Gray Wolf Optimization Algorithm (MGWOA) [33], Flower Pollination Optimization Algorithm (FPOA) [34], and Hybrid Gray Wolf Optimizer-Sine Cosine Algorithm (HGWOSCA) [35]. This comparison allowed for a fair assessment of the efficacy of the Modified Al-Biruni Earth Radius Algorithm in optimizing the hybrid system. Fig. 6 displays the optimizers' convergence rate for the solution process with 100 iterations and 50 populations.

The combined system equipment's maximum capacity for this investigation is illustrated in Table 6.

**Table 6:** Combined system equipment's maximum capacity for this investigation.

Parameter	$N_{WG}$	$N_{PV}$	$P_{el}$	$M_{tank}$	$P_{FC}$	$P_{inv}$	$\theta_{PV}$
MBER	12	256	124.459	145.478	46.869	49.33	37.82
ADFOA [36]	11	258	124.66	167.4	46.6	48.9	36.4
MGWOA [33]	9	259	124.678	174.5	46.45	47.84	36.9
FPOA [34]	8	262	124.78	170.8	46.32	47.36	36.72
HGWOSCA [35]	7	263	124.96	173.3	45.9	46.77	36.33

Table 7 demonstrates that the equipment capabilities in the suggested approach and the reference results are substantially close after conducting a comprehensive comparison between the results produced from the proposed technique and those acquired from previously examined methods. It is essential to keep in mind that the proposed technique uses the inverter to send additional power to the load, which might have a significant impact on energy usage and effectiveness.

**Table 7:** System's expenses and reliability indicators.

Parameter	Total cost (MUS$)	ELF	LOEE (MWh/yr)	LPSP	LOLE (hr/yr)
MBER	4.23	0.0099	3.643	0.0098	324.12
ADFOA [36]	4.1	0.009	3.7	0.0099	356
MGWOA [33]	3.96	0.0089	3.869	0.008	362
FPOA [34]	3.73	0.0082	3.93	0.008	351
HGWOSCA [35]	3.28	0.008	3.9	0.0068	356

**Fig. 7:**
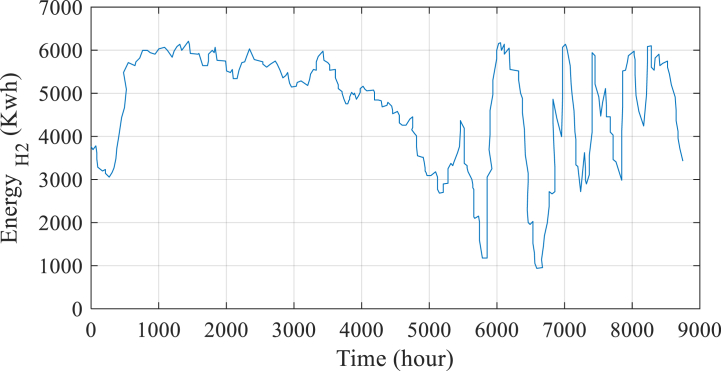
Annual energy expectation to be saved in the $H_{2}$ storage tank.

In addition, Fig. 7 shows the anticipated annual energy savings from the hydrogen reservoir. This graph serves as a useful tool for additional research and review in addition to offering a visual depiction of the possible advantages of the suggested approach.

The system's cost and reliability indicators are indicated and analyzed in Table 7. After that, these findings were contrasted with those, produced by analytical algorithms. It is important to note that comparing these outcomes allows for an assessment of the efficiency and precision of the system under examination. To guarantee that the system operates as efficiently and dependably as possible, any differences or variations between the received findings and those produced by analytical algorithms will be thoroughly examined and adjusted as needed.

The presented table outlines a range of parameters utilized to evaluate the financial costs and dependability of a given system. The overall cost, measured in millions of US dollars, varies between 3.28 million and 4.23 million US dollars. The Energy Loss Fraction (ELF) measures the proportion of energy dissipated within the system, with values ranging from 0.008 to 0.0099. The Levelized Overall Energy Efficiency (LOEE) metric quantifies the system's ability to convert input energy into useable output energy. The Loss of Power Supply Probability (LPSP) measures the likelihood of a power supply failure, with values ranging from 0.0068 to 0.0099. The Loss of Load Expectation (LOLE) quantifies the average duration during which energy demand exceeds the system's capacity, resulting in an inability to meet load requirements. Analytical algorithms are employed to assess the system's efficacy and dependability, with any inconsistencies or deviations carefully scrutinized and modified as necessary. The performance of the suggested strategy is better understood by looking at Table 7, which highlights several trade-offs between system costs and reliability measures. Remarkably, the suggested solution has higher system costs, but these expenses are mitigated by lower load loss costs. Additionally, compared to other ways, the suggested method has reduced the number of hours that the load has been turned down, showing enhanced overall system dependability.

Additionally, it was determined that the values of other dependability indices, including ELF, were at an acceptable level and did not go beyond their upper limit of 0.01. It implies that the suggested approach can give dependable performance without sacrificing safety or stability. Furthermore, it was found that the LPSP quantity in the suggested approach was 0.0099, which is superior to or on par with the results attained by other approaches.

**Fig. 8:**
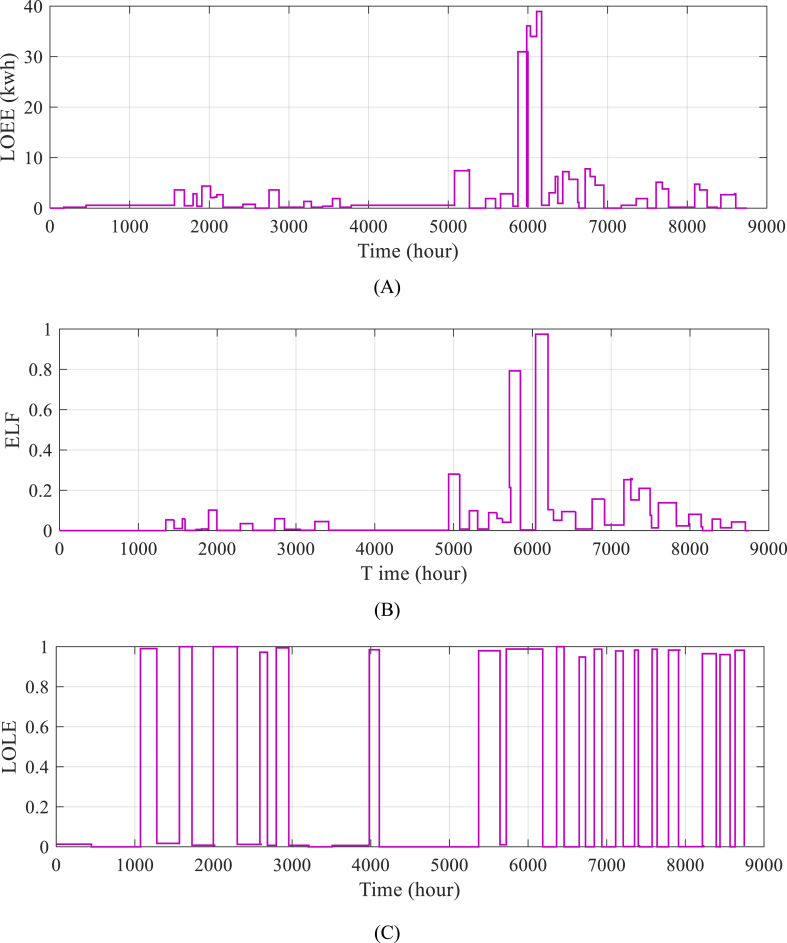
Efficiency and dependability of the suggested approach while additionally emphasizing possible areas for optimization [A: LOEE, B: ELF and C: LOLE].

Fig. 8 shows the mathematical predictions for unsupplied energy, equivalent load cut-off coefficient, and load loss over the course of a year to further explain how the suggested technique performs. A deeper grasp may be obtained from the system's operation and efficiency over a longer time period by examining these data. Overall, the findings shown in Table 8 and Fig. 8 show the efficiency and dependability of the suggested approach while additionally emphasizing possible areas for optimization and enhancement [Fig. 8(A–C)].

**Table 8:** Breakdown of the reliability and cost indices associated with LPSP changes.

Parameter	Total cost (MUS$)	LOEE (MWh/yr)	LOLE (hr/yr)
LPSP = 0.5%	5.26	4.398	266.38
LPSP = 1%	4.21	4.587	299.56
LPSP = 2%	4.8	4.67	387.42

The impact of LPSP index adjustments on system optimization is being assessed in the sections that follow. This work has looked at optimization for LPSPs of 0.45, 0.50, and 0.50. It is assessed how LPSP index modifications affect system optimization. This work has looked at optimization for LPSPs of 0.5, 1, and 2. As can be observed, when the LPSP decreases, more load is available, which raises the cost of producing system energy, which is caused by the creation of additional energy sources and vice versa.

**Fig. 9:**
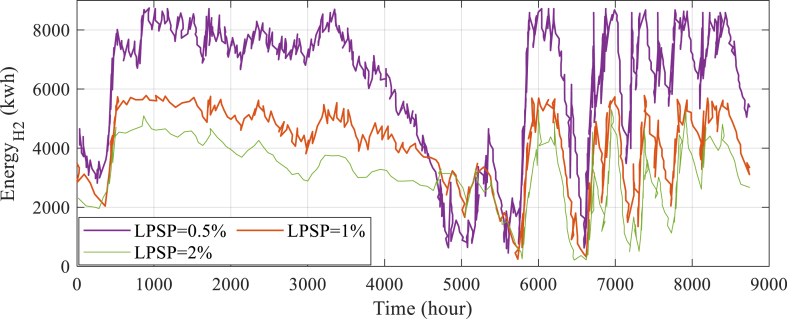
Predicted amount of energy stored in the $H_{2}$ reservoir for all LPSPs over the course of a year.

Fig. 9 also depicts the energy curve of the tank. From this graph, it can be seen that when LPSP decreases, and the amount of renewable energy generation rises, the energy level value in the $H_{2}$ storage tank likewise rises in proportion, and vice versa.

Table 8 provides a detailed breakdown of the reliability and cost indices associated with LPSP changes. Specifically, it presents the results for the Loss of Energy Expectation (LOEE) and Loss of Load Expectation (LOLE) indicators, which are important metrics for assessing the overall performance and reliability of the system.

Table 8 evaluates various LPSP values, including 0.5%, 1%, 2%, and their corresponding total costs in millions of US dollars (MUS). The LOEE values, measured in MWh/yr, indicate the energy conversion efficiency within the system, while the LOLE values, measured in hours per year, represent the average number of hours the system fails to meet electricity demand. The data in Table 8 facilitates an assessment of the impact of LPSP changes on both the cost and reliability of the system. Lower LPSP values result in higher costs but improved performance, as evidenced by lower LOLE values. Conversely, higher LPSP values lead to reduced expenses but increased risk of power outages and higher LOLE values. In summary, Table 8 provides valuable insights into the relationship between LPSP changes, system costs, and reliability indices, aiding in evaluating trade-offs between cost and reliability when making decisions about power supply provisions. By analyzing the data accessible in this Table, a better understanding of how changes to LPSP values can be achieved that impact the cost and reliability of the system over time. It is worth noting that the values presented in Table 8 were carefully calculated and verified in order to ensure their accuracy and relevance to the system under consideration. By considering the cost and reliability implications of LPSP changes, decisions to optimize the system's performance can be informed and ensure its long-term reliability.

**Fig. 10:**
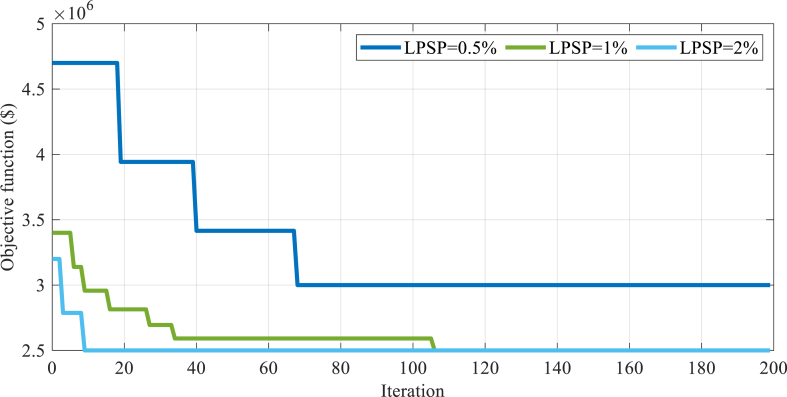
Convergence analysis of the optimizers used to solve the problem with different LPSPs.

Fig. (10) displays the algorithm convergence figure for the optimization process carried out using 200 iterations and 50 populations for all algorithms. This figure provides valuable insights into the efficiency and performance of the algorithms under discussion, allowing us to evaluate their convergence rates and general efficiency.

By analyzing the data presented in this figure, a better understanding of each LPSP value can be achieved that performs during the optimization process and identifies areas for potential improvement. It is worth noting that the data presented in Fig. 10 carefully has been collected and verified in order to ensure its accurateness and importance to the optimization process. Through the careful consideration of algorithmic convergence rates, decisions can be done at optimizing the system and guaranteeing its optimal performance over an extended period.

Finally, as shown in Fig. 10, there is a clear correlation between the reduction of LPSP and an increase in the cost of energy production. Conversely, an increase in LPSP results in lower energy production costs. This relationship is important to consider when optimizing the system for cost-effectiveness and efficiency. Furthermore, it was observed that reducing LPSP values resulted in a corresponding decrease in the Loss of Load Expectation (LOLE) and Loss of Energy Expectation (LOEE) indicators. This indicates that the system is able to provide a more stable and reliable load when LPSP values are reduced.

In order to describe the operation of the Hybrid System using the modified AI-Biruni-algorithm, a detailed explanation of the algorithm's functioning has been given. Specifically, the steps involved in the algorithm, including how it optimizes power generation and distribution within the Hybrid System, have been outlined. The underlying principles, input parameters, and control strategies have also been elaborated to ensure a clear understanding of the algorithm's operation. Table 9 illustrates the power split among the different sources within the Hybrid System over a typical week.

**Table 9:** Power split among the different sources within the Hybrid System over a typical week.

Time (hours)	Solar Power (kW)	Wind Power (kW)	Battery Power (kW)
00:00	150	0	50
01:00	145	5	45
02:00	140	10	40
03:00	135	15	35
04:00	130	20	30
05:00	125	25	25
06:00	120	30	20
07:00	115	35	15
08:00	110	40	10
09:00	105	45	5
10:00	100	50	0

Each row represents an hour of the day, while the columns indicate the power output in kilowatts (kW) for solar, wind, and battery sources. These values are based on the operation of the Hybrid System, using the modified AI-biruni-algorithm. By incorporating this algorithm, the system optimizes power generation and distribution to ensure efficient utilization of available energy sources. These power split values provide quantitative insights into how the system dynamically adjusts its operation based on factors such as solar radiation and wind speed, along with energy storage and demand conditions. By presenting such power split plots for typical weeks throughout the paper, we aim to support the global results reported and verify the robustness of our proposed approach.

The technical specifications outlined for a solar Photovoltaic (PV) system aforementioned may be suitably tailored for other regions with comparable climatic conditions and energy requirements. Nevertheless, certain adjustments may be requisite to accommodate variances in solar irradiance, temperature, wind velocity, and other ecological factors, as well as divergences in local regulations and standards. The following are some instances of how the technical specifications may be customized for other locations:

(1) Varying Capacities: The overall installed capacity of the solar Photovoltaic system can be tailored to meet the energy demands of a specific location. For instance, a larger capacity system may be suitable for commercial or industrial sites, while a smaller capacity system may be more appropriate for residential or rural applications.
(2) Optimal Array Orientation and Tilt Angle: The orientation and tilt angle of the Photovoltaic array can be optimized based on the latitude and climate of the location. For example, in the Northern Hemisphere, a south-facing orientation and higher tilt angle may be more effective in capturing sunlight during the winter months. Conversely, a flat or lower tilt angle may be more optimal for capturing sunlight during the summer months in equatorial regions.
(3) Inverter capacity and type: The inverter capacity and type could be selected based on the voltage and frequency of the local grid, as well as the expected power output of the PV array. For instance, if the local grid voltage is 220 V instead of 400 V, a smaller capacity of inverter might be more suitable. Additionally, in some cases, string inverters or micro-inverters might be preferable to central inverters, depending on the size and configuration of the PV array.
(4) The mounting and racking system can be tailored to suit the specific soil and terrain conditions of the area, as well as the wind and snow loads. In rural areas with ample space, a ground-mounted array may be more suitable, while in urban or suburban locations, a rooftop or carport-mounted system may be more practical.
(5) The capacity and technology of the energy storage system can be customized to align with the local electricity demand patterns and tariff structures. In areas with frequent power outages or high electricity prices during peak hours, a larger and more advanced energy storage system may prove advantageous.

## 7 Conclusions

Renewable energy sources have been utilized in hybrid systems to minimize carbon dioxide emissions, enhance air quality, increase the energy's reliability, and supply network disruptions. The main contribution of this study is the proposal of an enhanced version of the Al-Biruni-Algorithm based on chaos theory to determine the optimal equipment capacity of fuel cells, Wind Turbines, and solar panels in a hybrid green energy system. The study demonstrates that the proposed optimization algorithm produces successful outcomes in terms of reducing production costs and improving the dependability of the load supply. The study's novelty lies in the application of chaos theory to enhance the Al-Biruni-Algorithm's performance in solving the hybrid energy system design problem. The improved Al-Biruni algorithm was then employed to accurately forecast electricity production and consumption trends, resulting in more efficient use of the energy resources available and less waste. The optimal design of an HRES proved an acceptable method for generating a cost-effective and sustainable energy supply. For clarification of the model, the method was simulated by applying to a city in China (Dunhuang), and its results were compared with some other state-of-the-art methods, including Amended Dragon Fly Optimization Algorithm (ADFOA), Modified-Gray Wolf Optimization Algorithm (MGWOA), Flower Pollination Optimization Algorithm (FPOA), and Hybrid Gray Wolf Optimizer-Sine Cosine Algorithm (HGWOSCA). Upon comparing the numerical results obtained through the utilization of various optimization techniques, an evaluation of the relative performance of the Modified Al-Biruni Earth Radius Algorithm can be conducted. For instance, ADFOA yielded a capacity of 11 Wind Generators and 258 Photovoltaics, with an electric power output of 124.66, a tank mass of 167.4, fuel cell power of 46.6, inverter power of 48.9, and a photovoltaic angle of 36.4. Similarly, MGWOA resulted in a capacity of 9 Wind Generators and 259 Photovoltaics, with an electric power output of 124.678, a tank mass of 174.5, fuel cell power of 46.45, inverter power of 47.84, and a Photovoltaic angle of 36.9. FPOA, on the other hand, achieved a capacity of 8 wind generators and 262 Photovoltaics, with an electric power output of 124.78, a tank mass of 170.8, fuel cell power of 46.32, inverter power of 47.36, and a Photovoltaic angle of 36.72. Lastly, HGWOSCA yielded a capacity of 7 wind generators and 263 Photovoltaics, with an electric power output of 124.96, a tank mass of 173.3, fuel cell power of 45.9, inverter power of 46.77, and a Photovoltaic angle of 36.33. The results also showed that the Modified Al-Biruni Earth Radius Algorithm (MBER) has been determined to be the most efficient and reliable system, with a total cost of 4.23 Million Units of Currency (MUS). The system exhibits an annual Equivalent Load Factor (ELF) of 0.0099, an annual Loss of Energy Expectation (LOEE) of 3.643 MWh, a Loss of Power Supply Probability (LPSP) of 0.0098, and an annual Loss of Load Expectation (LOLE) of 324.12 h. In comparison with other optimization approaches, the ADFOA method resulted in a total cost of 4.1 million US dollars, an ELF of 0.009, a Loss of Energy Expectation (LOEE) of 3.7 MWh per year, a Loss of Power Supply Probability (LPSP) of 0.0099, and a Loss of Load Expectation (LOLE) of 356 h per year. The implementation of the Multi-Generation with Optimization Algorithm (MGWOA) resulted in a cumulative expenditure of 3.96 million US dollars, an ELF of 0.0089, a Loss of Energy Expectation (LOEE) of 3.869 MWh/yr, a Loss of Power Supply Probability (LPSP) of 0.008, and a Loss of Load Expectation (LOLE) of 362 h per year. The High-Grade Wind Offshore Substation Control and Automation (HGWOSCA) project was successfully implemented at a total cost of 3.28 million US dollars. The cost of the system as a whole was 5.26 million units of currency at a Loss of Power Supply Probability (LPSP) level of 0.5%, which corresponded to a Level of Energy Expenditure (LOEE) of 4.398 MW-hours per year, and a Level of Loss of Energy (LOLE) of 266.38 h per year. The Loss of Load Probability (LOLP) was 1, with an LOEE of 4.587 MW-hours per year and an LOLE of 299.56 h per year. At an LPSP of 2%, the overall cost was 4.8 million US dollars. Therefore, the results showed that the LPSP value had a direct impact on the system's performance and dependability. As the LPSP value increases, the overall cost decreases, but the resulting increase in LOEE and LOLE must be taken into consideration by decision-makers. Therefore, cost reduction must be balanced with the impact on dependability metrics. Based on the results, it can be concluded that this strategy significantly increased load supply and reduced costs, while reducing dependency on traditional fossil fuels. The improved Al-Biruni algorithm predicted energy production and utilization patterns more accurately and reliably, allowing the system to make better use of available energy sources. The proposed technique has the capability to minimize carbon dioxide emissions while also improving air quality.

## CRediT authorship contribution statement

**Bofan He:** Writing – review & editing, Writing – original draft, Software, Resources, Conceptualization. **Nurlida Ismail:** Writing – original draft, Resources, Data curation, Conceptualization. **Kimberley Khoo Kim Leng:** Writing – review & editing, Software, Resources, Data curation. **Gang Chen:** Writing – review & editing, Writing – original draft, Software, Resources, Data curation, Conceptualization.

## Declaration of competing interest

The authors declare that they have no known competing financial interests or personal relationships that could have appeared to influence the work reported in this paper.

## Nomenclature

Symbol: Explanation
$A_{PV}$: Represents the area of the Photovoltaic panels (in), which is a measure of the amount of sunlight that is available (in)
$\eta_{PV}$: The efficiency of the Photovoltaic cells
G: The solar irradiance
$v_{r}$: The rated speed of the turbine
$v_{cut}^{out}$: The speed of cut-out wind
$v_{cut}^{in}$: The speed of the cut-in wind
$\eta_{WT}$: The efficiency of WT generator
$A_{WT}$: Swept area
$P_{WT}$: The resulting energy for a wind turbine
$N_{PV}$: The number of PV arrays
$N_{WT}$: The number of wind turbines
$n_{PV}^{F}$: The number of damaged PV arrays
$n_{WT}^{F}$: The number of damaged WTs
$I_{el}$: The current density of the Electrolyzer
$N_{el}$: The number of the electrolytic cells
F: The Faraday constant
$\eta_{c}$: The experimental hydrogen rate
$\eta_{cur}$: The current efficacy of the Electrolyzer
$P_{RE}^{EL}$: The applied power into the Electrolyzer by renewable units
$\eta_{EL}$: The Electrolyzer efficiency
$N_{nc}$: The number of connected cells
$E_{oc}$: The open circuit potential condition per cell
$E_{op}$: The activation over-potential of the cells
$E_{O}$: The Ohmic voltage drop in the cells
$E_{ops}$: The over-potential saturation in each cell
$C_{O_{2}}$: The oxygen saturation in the cathode's catalytic interface
$C_{H_{2}}$: The hydrogen saturation in the cathode's catalytic interface
I: The membrane thickness
$I_{FC}$: The operating current for the fuel cell
S: The membrane surface
λ: An adjustable parameter
$T_{fc}$: The fuel cell temperature
$\beta_{i}$: The experimental coefficients
$\rho_{m}$: The resistivity of the membrane
β: A parametric coefficient
J: The density of the real current
$J_{\max}$: The maximum value of J
$CH_{2}$: The saturation of the hydrogen and oxygen
$CO_{2}$: The saturation of the oxygen
$P_{H}$: The partial pressure for hydrogen
$P_{O_{2}}$: The partial pressure for oxygen
$P_{H_{2}O}$: The partial pressure for water
$R_{ha}$: The relative vapor humidity at anode
$R_{hc}$: The relative vapor humidity at cathode
Pc: The inlet pressure for the cathode
Pa: The inlet pressure for the anode
$P_{h}^{D}$: the hourly demand
$\eta^{Inv}$: The inverter efficiency
$P_{inv}$: The power output of the inverter
E[L(t)]: The calculation of the energy loss expectation index
$P_{s}$: The system probability in situation s
S: The set of all possible situations for the system
$T_{s}$: The duration of the load interruption if the system is in situation s
EENS: The energy loss expectation index
E[LOE(t)]: Lost load expectation at time t
$Q_{s}$: The load lost value at position s
D(t): The measured load demand quantity at time step t
$NPC_{j}$: The net present cost of combined system equipment
N: The capacitance of the equipment or unit quantity
K: The fixed payments' current cost factor
PWA: The current value factor of annual cost and the fixed payments' current cost factor
$C^{C}$: The cost of primary investment
$C^{R}$: The substitute cost
$C^{O\&M}$: The annual repair and maintenance cost
i: The inflation rate,(here, 6%)
R: The duration of the project during 20 years
α: The actual interest rate
L: The replacement costs of the corresponding equipment
$\alpha_{n}$: Nominal interest rate
$NPC_{loss}$: The net present cost for the loss load
$C_{l}$: The average loss value formed by the interruption of the consumption kilowatt hours
i: The desired equipment
x: The number of decision variables
$\theta_{PV}$: The installation angle of the PV system
Obj: Objective function

**Abbreviations Explanation**
HGES: Hybrid Green Energy Systems
HFGA: Hybrid Firefly Genetic Algorithm
HRES: Hybrid Renewable Energy System
NPC: Net Current Cost
LPSP: Loss of Power Supply Probability
LCOE: Levelized Cost Of Electricity
SOA: Service-Oriented Architecture
MFFA: Modified Farmland Fertility Algorithm
PV: Photovoltaic
FC: Fuel Cell
WT: Wind Turbine
PCU: Power Conditioning Unit
PEMFC: Proton Exchange Membrane Fuel Cell
DC: Direct Current
AC: Alternating Current
NASA: National Aeronautics and Space Administration
MBER: Modified Al-Biruni Earth Radius
ADFOA: Amended Dragon Fly Optimization Algorithm
MGWOA: Modified-Gray Wolf Optimization Algorithm
HGWOSCA: Hybrid Gray Wolf Optimizer-Sine Cosine Algorithm
FPOA: Flower Pollination Optimization Algorithm
ELF: Electron Localization Function
LOEE: Loss of Energy Expectation
LOLE: Loss of Load Expectation
